# Bacterial Involvement in Progression and Metastasis of Colorectal Neoplasia

**DOI:** 10.3390/cancers14041019

**Published:** 2022-02-17

**Authors:** Kevin D. Seely, Amanda D. Morgan, Lauren D. Hagenstein, Garrett M. Florey, James M. Small

**Affiliations:** 1 College of Osteopathic Medicine, Rocky Vista University, Ivins, UT 84738, USA; amanda.morgan@rvu.edu (A.D.M.); lauren.hagenstein@rvu.edu (L.D.H.); 2 College of Osteopathic Medicine, Rocky Vista University, Parker, CO 80134, USA; garrett.florey@rvu.edu; 3 Department of Biomedical Sciences, Rocky Vista University, Parker, CO 80134, USA; jsmall@rvu.edu

**Keywords:** gastrointestinal neoplasm, colorectal carcinoma, metastasis, carcinogenesis, malignancy, bacterial infection, infectious disease, epithelial–mesenchymal transition, infectious disease

## Abstract

**Simple Summary:**

Increasing evidence suggests that bacterial infection not only promotes carcinogenesis in primary colorectal cancer, but also affects metastatic progression and organ selectivity through modification of the microenvironment at primary and secondary tumor sites. The metastatic cascade is the process by which neoplastic tumors potentiate cancerous spread to distant organs, and evidence suggests that this process is provoked in the setting of bacterial infection. Biofilm formation, paired migration, and quorum sensing are processes by which bacteria self-signal, recruit, and effectively establish a pre-metastatic niche at distant sites, rendering a suitable environment for tumor cell survival and proliferation.

**Abstract:**

While the gut microbiome is composed of numerous bacteria, specific bacteria within the gut may play a significant role in carcinogenesis, progression, and metastasis of colorectal carcinoma (CRC). Certain microbial species are known to be associated with specific cancers; however, the interrelationship between bacteria and metastasis is still enigmatic. Mounting evidence suggests that bacteria participate in cancer organotropism during solid tumor metastasis. A critical review of the literature was conducted to better characterize what is known about bacteria populating a distant site and whether a tumor depends upon the same microenvironment during or after metastasis. The processes of carcinogenesis, tumor growth and metastatic spread in the setting of bacterial infection were examined in detail. The literature was scrutinized to discover the role of the lymphatic and venous systems in tumor metastasis and how microbes affect these processes. Some bacteria have a potent ability to enhance epithelial–mesenchymal transition, a critical step in the metastatic cascade. Bacteria also can modify the microenvironment and the local immune profile at a metastatic site. Early targeted antibiotic therapy should be further investigated as a measure to prevent metastatic spread in the setting of bacterial infection.

## 1. Introduction

Cancer is among the leading causes of death worldwide and is the second leading cause of death in the United States [1,2]. Gastrointestinal (GI) cancers, including colorectal cancer (CRC), account for approximately one-third of total global cancer incidence and mortality [3]. Not only are they common, but metastatic GI tumors have high mortality rates, rendering these malignancies among the most prominent public health issues of our time [4]. As the third most common malignancy and the second most deadly, colorectal cancer (CRC) has an estimated incidence of 1.9 million cases and caused 0.9 million deaths worldwide in 2020 [5]. Metastatic disease contributes to more than 90% of cancer-related deaths and remains a major clinical challenge in oncology [6]. If identified in an early stage, primary tumors can often be controlled with local surgery or radiation. Unlike primary tumors, however, metastatic cancer is a systemic disease, which requires systemic approaches to treatment [6]. 

It has been proposed that some bacterial species, referred to in the literature as microbial drivers, carry genes encoding proteins that can induce chromosomal instability and initiate the oncogenic cascade in intestinal epithelial cells [7,8]. Other opportunistic bacteria, referred to as microbial passengers, could become more prevalent in a pre-tumor microenvironment, boosting inflammation, and fostering oncogenesis or cancer progression [8,9]. Although much has been written about the relationship between certain bacteria and oncogenesis, the literature pertaining to bacteria associated with metastatic disease is limited, and understanding of whether or not a tumor is dependent upon the same microbial microenvironment during or after hematogenous or lymphatic spread is in its infancy. The idea that certain microbial drivers might be associated with not only primary tumors but also their metastases is a relatively novel concept. The interrelationship between specific bacteria and CRC is well studied, rendering this particular entity an ideal model for considering the idea of bacterial drivers of metastasis.

We first review CRC progression and metastasis in Section 2. The current literature on bacterial involvement in CRC is reviewed in Section 3, followed by analysis of the literature to discover the role of bacteria in CRC metastasis and organotropism and the microenvironment of both primary and secondary sites of proliferation in Section 4. Note that viral drivers and passengers are outside the scope of this review. Section 5 addresses tumor microenvironment considerations in treatment. Overall, we aim to build upon the existing knowledge about the role of bacteria in cancer progression and metastatic disease by reviewing the current literature pertaining to bacterial drivers of CRC and their effect on metastasis to identify new exploitable points of intervention.

## 2. Colorectal Neoplasia and Metastasis

As with other anatomic sites, the entire gastrointestinal tract may undergo neoplastic transformation [10]. Within each organ, each cell type, similarly, may transform. Neoplasms may arise in the esophagus, stomach, small intestine, large intestine, and anus. Within each organ, epithelial cells may give rise to carcinomas or adenomas, mesenchymal cells may give rise to benign or malignant tumors such as sarcomas or gastrointestinal stromal tumors, and lymphoid tissue may transform into lymphomas.

It is worth remembering, therefore, that a mass in the stomach, large intestine, liver, and so on has a differential diagnosis well beyond simply adenocarcinoma. The most common colonic tumors include adenomas, serrated tumors, adenocarcinomas, carcinoid tumors, lymphomas, and mesenchymal tumors.

Colorectal cancers have the potential to metastasize to sites including, but not limited to, lymph nodes, lungs, peritoneum, bone, spleen, and liver. Different metastasis patterns depend on the primary site and the type of cancer. In general, Budczies et al. [11] report that the most common distant metastasis sites for colorectal carcinoma are the liver, non-regional lymph nodes, lungs, and peritoneum. Qiu et al. [12] complement these findings by reporting that the most well-known metastatic sites for colorectal cancer are the liver and lung. It should be noted that spread to regional lymph nodes is far more common than distant metastasis, but is usually considered separately.

**Figure 1:**
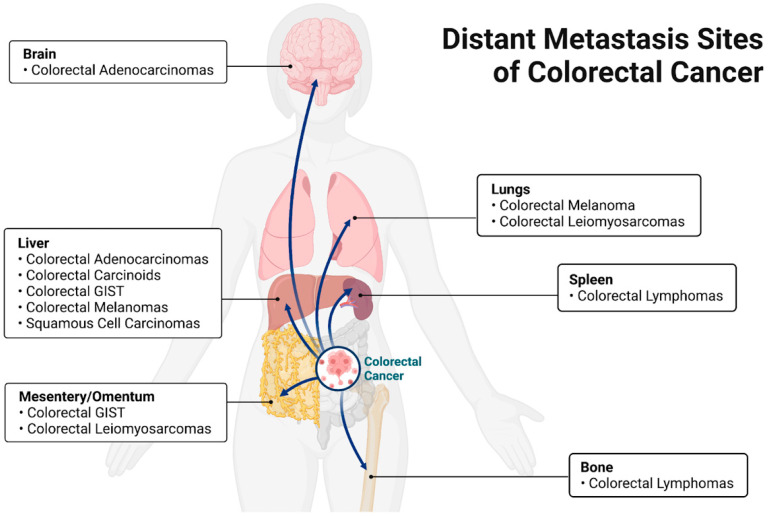
Most common sites of metastasis of colorectal neoplasia specific tumor type. Figure created using biorender.

Colorectal adenocarcinomas (CRC) and colorectal carcinoid tumors account for the majority of colon and rectum cancers. Colorectal adenocarcinomas metastasize to the liver most commonly (70%), followed by the lung (24%), distant lymph nodes (16%), and peritoneum (15%) [13]. Of note, colorectal adenocarcinomas have also been found to metastasize to the brain [14]. Colonic carcinoids, another major colon cancer, were found to mostly metastasize to the liver [15]. Some rare types of colorectal cancers include primary colorectal lymphomas, gastrointestinal stromal tumors (GIST), leiomyosarcomas, melanomas, squamous cell carcinomas, and familial adenomatous polyposis. Rectal GIST has been shown to spread intraperitoneally and to the liver [16]. Of special note, leiomyosarcomas metastasize to the lung and peritoneum [17]. Primary colorectal melanomas have been shown to spread to the liver and lung [18]. Finally, squamous cell carcinomas mainly metastasize to the liver [19]. The most common sites of distant metastasis from a colon carcinoma are the liver (62%), thorax (8%), and peritoneum (4%). Rectal cancers were found to commonly metastasize to the liver (61%), lung (19%), and bone (4%) (Figure 1, Table 1) [20]. This review considers only the pathogenesis of CRC and not the other neoplasms that may arise in the colon.

### 2.1. Carcinogenesis

Neoplastic transformation leading to adenocarcinoma in the colon is currently thought to occur via multiple inborn or acquired genetic mutations [22,23]. Errors in cell division and uncontrolled growth can be caused by genetic modifications which are inherited or acquired during a person’s lifetime [24]. Cancer formation is thought to be initiated by stem cell DNA damage that overrides DNA repair mechanisms, a specific and progressive form of genetic modification [25]. Carcinogens are agents that can induce this type of DNA damage, increasing the risk of cancer [26]. Radiation exposure [27], smoking and alcohol consumption [28], nutrition [29], inflammation [30], and infectious microbes such as viruses, bacteria, and parasites are among the various external causes that can produce these mutations [31]. DNA mutations that disrupt key regulatory systems modulated by proto-oncogenes [32], tumor suppressor genes [33], or regulators of apoptosis [34,35] allow for tumor growth and progression. While many individual tumors may arise sporadically without a clear-cut premalignant sequence in the large intestine, it is thought that many malignancies arise in a stepwise fashion through benign neoplastic intermediates in a process termed the adenoma–carcinoma sequence.

#### 2.1.1. Adenoma–Carcinoma Sequence

The classical pathway of colorectal carcinoma (CRC) development, also termed the adenoma–carcinoma sequence, is an umbrella term for two distinct mechanisms of carcinogenesis: chromosomal instability (CIN) and microsatellite instability (MSI) [36]. These two mechanisms are driven by the accumulation of genetic mutations that lead to the formation of a premalignant adenoma, whether tubular or tubulovillous. The adenoma–carcinoma sequence is the most common cause of CRC and is sporadic in 70–85% of cases [37,38,39]. Well-studied risk factors include obesity and metabolic syndrome, low-fiber intake, high-fat intake, high red meat intake, and male sex [39,40,41]. A poorly balanced diet is theorized to expose the gut to more bacteria that are inflammatory and promote the CIN and MIS pathways [42]. 

The CIN pathway usually starts with a mutation in the Adenomatous polyposis coli (APC) gene, which can occur through various mechanisms, such as two-point mutations or loss of heterogeneity [43]. This event leads to β-catenin stabilization, which promotes downstream WNT gene dysregulation. At this point, any of several other regulation genes such as KRAS [44] and TP53 [45] can undergo mutations and further promote carcinogenesis. If multiple lineages of tumors happen to have overlapping sites, it is thought that the line with the most favorable set of mutations for growth will out-compete the other clones and dominate the tumor. Cancer-specific tumor markers are important in determining prognostication and treatment planning [46]. 

The microsatellite instability pathway involves mutations that affect the ability of the mismatch repair mechanism to correct replication errors [47]. Accumulation of errors such as methylation or mutations in mismatch repair genes (MMR genes, e.g., MLH1 or MSH2) leads to DNA polymerase dysfunction [48]. Approximately 15% of CRCs occur by the MSI pathway, with the MLH1 protein usually being inactivated due to hypermethylation of its promoter [49,50]. High and low MSI mutations increase susceptibility to checkpoint inhibitor malfunction and have better and worse prognosis, respectively. While a full discussion on mechanisms of the high and low pathways are outside of the scope of this review, we refer the reader to a review on this topic conducted by Li et al. [51] for further inquiry. Identification of this pathway necessitates the need for familial genetic testing, as MSI is also a hallmark etiology of hereditary nonpolyposis colorectal cancer (HNPCC), also known as Lynch syndrome (LS), in addition to some sporadic colorectal cancers [50,52]. 

**Figure 2:**
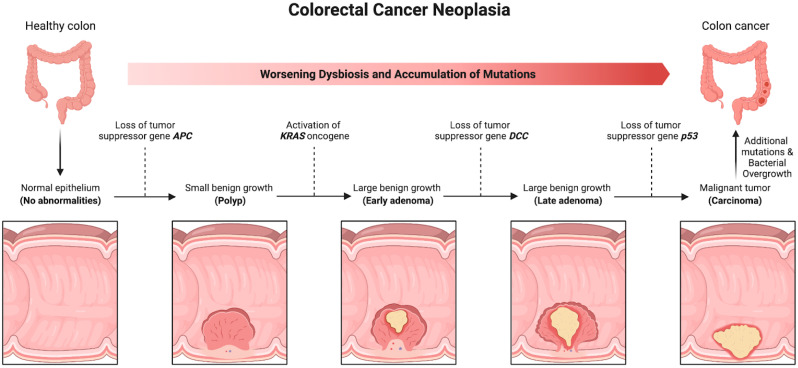
Adenoma–carcinoma sequence. Loss of tumor-suppressor gene, APC, results in hyperproliferative epithelium due to loss of cellular adhesion and increased proliferation. Further mutations of KRAS, delete in colorectal cancer (DCC), and p53 accumulate to yield carcinoma. Figure created using biorender.

As stated, this pathway is characterized by loss of DNA mismatch repair protein function. The dysfunction of DNA mismatch repair (MMR) enzymes, which is caused by germline mutations in one of several DNA mismatch repair genes, most commonly MLH1 or MSH2, is a key component of MSI. Due to the silencing of MMR genes, cells with a deficient DNA repair capacity accumulate DNA errors throughout the genome [53]. Characteristic of the MSI pathway is accumulation of microsatellites, which are abnormalities in short sequences of nucleotide bases that are repeated hundreds of times within the genome. These tumors are said to have high levels of microsatellite instability. Microsatellite instability has been recognized as a distinct mechanism promoting tumorigenesis in 3% of Lynch syndrome cases and 12% of sporadic CRCs [54]. In most cases of sporadic CRCs, however, gene silencing is not due to a specific MMR mutation but to hypermethylation of the gene promoter for the MMR enzyme (usually MLH1), which leads to transcriptional silencing of gene expression (Figure 2) [55,56].

#### 2.1.2. Serrated Neoplasia Pathway

While the majority of CRCs are thought to follow an adenoma–carcinoma pattern, recent evidence increasingly supports the possibility of an alternate route for colorectal carcinogenesis via serrated polyps, a morphological spectrum that includes hyperplastic polyps, mixed hyperplastic polyp/adenoma, and serrated adenomas in what is termed the serrated neoplasia pathway [57]. It is now thought that the serrated neoplasia pathway accounts for 30–35% of CRC [58]. The serrated neoplasia pathway of CRC development is driven by the CpG island methylator phenotype (CIMP) and is characterized by a high frequency of methylation of some CpG islands, otherwise termed the epigenetic instability pathway [36]. Via this method, hypermethylation of the CpG island foci inactivates tumor-suppressor genes and allows the formation of hyperplastic polyps and traditional serrated or sessile adenomas. Additionally, microsatellite instability may occur but is not required for CIMP to lead to unregulated growth. Right-sided CRCs are more associated with the CIMP pathway of origin, which may be due to different embryological origins [59]. Continuing to identify new routes of neoplasia such as this one will allow more opportunities to create more effective treatment plans.

### 2.2. Metastasis

Metastasis is the spread of cancer cells from the primary tumor to a distant organ or body tissue [60]. Cancer cells break away from the original (primary) tumor, travel through the blood or lymph system, and form a new tumor in other organs or tissues of the body in a process known as the metastatic cascade. The primary tumor and the new metastatic tumor originate from the same cell lineage. Invasion and metastasis are the major causes of cancer-related morbidity and mortality. Following the establishment of a malignant tumor at a primary site, there is significant potential for metastasis to distant regions of the body [61]. Tumor invasion and progression occur as a result of the accumulation of mutations [62]. 

In order to spread successfully, cancer cells must undergo a series of steps involving intricate adaptive reciprocity with host cells and factors in a process termed the metastatic cascade [63]. Individual genes directly implicated in metastasis have not been found, but several key mutations have been identified that increase metastatic potential [64]. At each step in the process of metastasis, breakaway cells must avoid or overcome the host immune system and adapt to a new microenvironment. This may be one area in which bacteria might have an effect [65,66]. Neoplastic cells must undergo a pattern of dissemination and invasion, intravasation, circulation, extravasation, and finally, colonization [63].

The local invasion, damage, or destruction of vital structures by tumor cells is a prerequisite for emergence from the primary mass and distant spread [67]. This process appears to be mediated by a network of cellular adhesion molecules, including E-cadherin and β-catenin [68]. Invasion alters the structural organization and function of normal tissues, loosening intercellular junctions and degrading and remodeling the extracellular matrix, and allows for the migration of tumor cells [66]. Alterations in intercellular adhesion molecules, such as E-cadherins, lead to the dissociation of cancer cells from one another. 

E-cadherins are transmembrane glycoproteins that mediate intercellular adhesion and signaling [69]. Several epithelial tumors, including adenocarcinoma of the stomach involve diminished E-cadherin function due to pathogenic mutations [70]. Cellular adhesion molecules such as E-cadherin and β-catenin maintain epithelial tumor cell connections in the primary tumor. E-cadherin functions as a tumor suppressor, and its loss is associated with advanced tumor stage and poor prognosis [71]. The E-cadherin/β-catenin complex helps maintain epithelial integrity. Disrupting this complex affects cell–cell adhesion and the Wnt-signaling pathway [68,72]. Dissociation of attached cells occurs when E-cadherin is downregulated, inhibited, or destroyed. 

It is hypothesized that E-cadherin expression is silenced in some cancers through epithelial–mesenchymal transition (EMT), a process in which epithelial cells are converted to full mesenchymal phenotypes [73,74,75]. The complex biological process of EMT has been determined to be a defining feature of carcinogenesis, as EMT-derived tumor cells develop stem cell characteristics, multiply rapidly, and are highly resistant to treatment [76]. Multiple kinase-mediated signaling pathways contribute to EMT and metastasis, some of which are initiated by bacterial infection. For example, *Enterococcus faecalis* has been implicated in the TGFβ-1/Smad signaling pathway in the setting of IL-10 deficiency in murine studies [77]. 

Changes in tumor cell attachment to the extracellular matrix (ECM) and locomotion of tumor cells constitute the final steps in the metastatic cascade [78]. Normal cells undergo a programmed cell-death called anoikis after dissociating from the ECM [79,80]. Tumor cells, on the other hand, are resistant to this form of cell death due to the expression of integrins that maintain adhesion to the ECM, enabling ongoing signaling that promotes cell survival [81,82]. Propulsion of tumor cells through the altered basement membrane and zones of matrix proteolysis constitutes the final step of the cascade [83]. Tumor cell locomotion is a multistep process involving many receptors and signaling pathways, all of which eventually interact with the actin cytoskeleton. In order to move forward, cells must attach to the matrix at their leading edge, detach from the matrix at their trailing edge, and contract the actin cytoskeleton [84]. 

**Figure 3:**
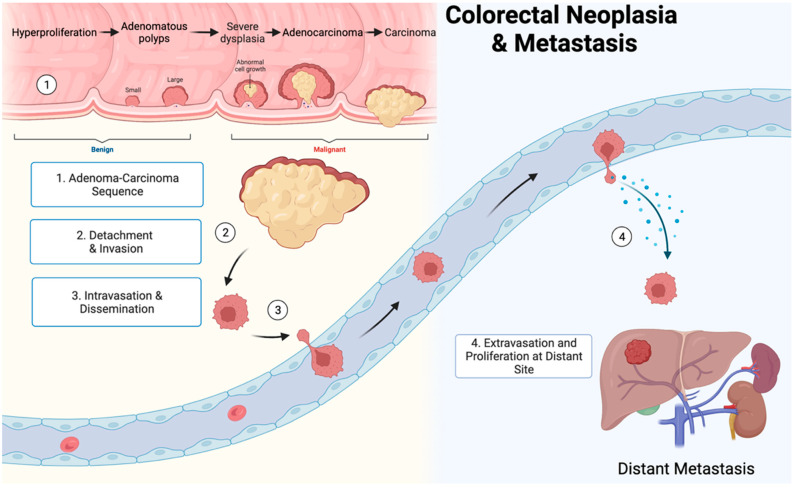
The metastatic cascade is a stepwise process involving carcinogenesis, detachment, and invasion, intravasation and dissemination, and extravasation and proliferation at a distant site. Figure created using biorender.

Tumor cell-derived cytokines, including chemokines and growth factors, act as autocrine motility promoters [85] and stromal cell-derived paracrine factors stimulate motility locally [86]. Collagenase is used by cancer cells to attach to laminin and destroy basement membrane collagen type IV [87]. Cells then attach to fibronectin in the extracellular matrix and spread locally [88]. Entrance into vascular or lymphatic spaces allows for distant spread [89]. New and evolving research indicates the potential for malignant gastrointestinal tumors to spread through mechanisms such as perineural invasion [90]. However, in the setting of colorectal cancers, two of the most common pathways for metastasis are through the lymphatic and venous systems [89]. The metastatic cascade culminates in penetration through the endothelial basement membrane and transmigration into lymphatic and vascular spaces (Figure 3).

#### 2.2.1. Lymphatic Spread of Colorectal Neoplasia

CRC can spread through both the lymphatic system and venous system to distant regions of the body. The lymphatic system is a complex series of connected channels, organs, and lymph nodes that allows for the drainage of fluid to maintain fluid balance, present antigens, limit bacteria, and facilitates tumor spread [91,92,93]. Various carcinomas metastasize to local and distant organs and regions of the body through the lymphatic system [94]. Often, lymph node metastasis is used to stage cancer as seen through the use of the TNM staging system, where N represents the spread of cancer to adjacent lymph nodes [95]. Through this system, physicians are able to determine the prognosis and precise treatment protocols of specific cancers. Lymphatic spread is mediated by the lymph node microenvironment, which may establish ideal conditions for the metastasis to take place [94,96].

One study demonstrated that colon cancer commonly metastasizes to the liver, thorax, and peritoneum [20]. Naxerova et al. [97] found that many distant organ metastases, such as those found in the liver, can be traced genetically back to a lymph node metastasis, as seen in 35% of the patient cases. The remaining 65% illustrated that distant metastases originated from both the primary tumor and lymph node lesions, therefore indicating that lymph nodes have some role in the metastatic cascade of primary colon tumors. However, the exact extent of its function is unknown.

Metastasis of colorectal tumor cancer cells may initially occur through epithelial–mesenchymal transition (EMT) [73,74]. EMT is a reversible process, through mesenchymal–epithelial transition (MET), which once undergone allows cells to colonize new areas of the body [98]. Once carcinoma cells metastasize through EMT and migrate to distant sites, they need to adapt to their new environment. The ability of cells to survive in their new environment has been postulated to be due to cell fusion, a process in which multiple cells combine to produce multinucleated cells with new properties to support cell adaptation and survival [99].

The precipitating factor which leads to EMT is not fully known. Chen et al. [100] found that when colon cancer cells are exposed to GM-CSF there is overexpression of EMT. It is hypothesized that this occurs through MAPK/ERK-ZEB1 signaling pathways. In cells with high GM-CSF, there was a significant correlation to lymph node metastasis. This gives important insight into the potential initiator of EMT and its ability to facilitate spread to metastasis sites via the lymphatic system. Another study illustrates that both TFF3 (secretory peptide) and TWIST1 (peptide and transcription factor), were found in CRC and correlated with increased potential for lymph node metastasis. Additionally, TTF3 may correlate with EMT [101]. Genes such as caspase-3, AHA1, CENPI, CTNNB1, and various long non-coding RNAs appear to be upregulated in CRC and could contribute to the EMT process [102,103,104,105,106]. Overall, there seems to be little consensus on a precise mechanism for how CRC undergoes EMT and metastasis via the lymphatic system to distant sites. Further research needs to be conducted to determine the precise mechanism of action and the greater role EMT plays in CRC metastasis.

#### 2.2.2. Venous Spread of Colorectal Neoplasia

Since the 1930s, it has been established that CRC metastasis occurs through the venous system [107,108]. However, this method of CRC metastasis has not fully been explored in terms of its ability to be utilized in a clinical setting and predict future prognosis [108]. Leijssen et al. [109] explored intramural and extramural vascular invasion (IMVI and EMVI) in colon cancer. Their study indicated that there was a strong correlation between EMVI and recurrence of colon cancer. EMVI was also shown to be indicative of future prognosis and mortality from Stage II-III colon cancer. Dirschmid et al. [110] study supported these findings and further expanded them to pertain to patients with colorectal neoplasia. This study also found that the presence of EMVI presented an increased risk for Stage I CRC to metastasis later in the disease course.

Current research is focused on understanding the role of venous spread in the metastatic cascade of colorectal carcinoma through both circulating tumor cells (CTCs), tumor cells that circulate in the blood, or circulating tumor DNA fragments (ctDNA) [6,111]. There are various hypotheses to further explain the mechanism of how tumor cells spread from the primary tumor including EMT or CTC clusters. CTC clusters are larger groups of cells that break off from the primary tumor and can spread cancer to distant sites or may stay in the capillaries [112]. Overall, CTCs are found to circulate in patients with malignant tumors, such as colorectal cancers, and thus have the potential to become a common test to assess cancer tumors [113,114]. These CTC biomarkers have been shown to correspond with the overall prognosis and severity of the disease based upon the remaining CTC biomarkers present in the bloodstream after cancer treatment [115]. CTC biomarkers have already been established as a method to monitor other carcinomas such as breast cancer [114]. Preliminary studies indicate that CTCs found in peripheral blood correlates to worse prognosis in patients with CRC [116,117].

#### 2.2.3. Transcoelomic Spread

Seeding of body cavities is typically considered a characteristic of ovarian [118], gastric [119], pancreatic [120], and colorectal carcinoma [121] and often involves the peritoneum [122]. Lung cancer also commonly seeds parietal pleura and thoracic cavity [123]. Peritoneal metastasis is one of the major indications of unresectability in colorectal cancer and a cause of death in advanced CRC [124]. Identification of distinct gene expressions between primary CRC and peritoneal seeding metastasis has been proven helpful in predicting the metastatic potential of primary human CRC [121]. 

A study using surgically implanted mouse ovarian cancer cells into the oviducts of syngeneic mice and simulated conditions associated with ovulatory wound repair, incessant ovulation, ovarian surface scarring, and aging sought to determine which conditions are conducive to the seeding of cancer cells in an immunocompetent mouse model. Not the ovary, but a nearby surgical wound site, which was associated with a strong and persistent inflammatory response, was found to be the most common site of cancer cell seeding [118]. The histone demethylase KDM4B has been found to regulate seeding and growth of peritoneal tumors in vivo, where its expression corresponds to hypoxic regions [125].

## 3. Bacterial Involvement in Colorectal Neoplasia Progression

The ability of certain microorganisms to induce oncogenesis has been widely studied [126,127,128]. In total, 2.2 million (13%) of new cancer cases are attributable to 10 carcinogenic pathogens according to data from the Global Cancer Observatory (GLOBOCAN). These carcinogens are further subclassified by the International Agency for Research on Cancer (IARC) as six viruses (Epstein–Barr virus, human papillomavirus, hepatitis virus B, hepatitis virus C, human herpesvirus type-8, and human T-cell lymphotropic virus type-1), one bacterium (*Helicobacter pylori*), and three parasites (*Opisthorchis viverrine, Clonorchis sinensis,* and *Schistosoma haematobium*). Chronic immunosuppressive infections such as human immunodeficiency virus (HIV) are excluded from these criteria, although a secondary effect of HIV is the increase in the carcinogenicity of viruses and bacteria [129]. *H. pylori* was classified by the World Health Organization (WHO) as a Class A carcinogen in 1994 [130] and remains the only bacterium to be classified as a known carcinogen at the time of this writing. 

An important delineation to make is between association and causation. Numerous bacteria have documented associations with certain cancers. However, this does not always mean they are causal. As previously stated, *H. pylori* remains the only bacterium classified by the WHO as a class A carcinogen. However, vast research on cancer-causing or cancer-associated bacteria has discovered multiple species that have complex interrelationships with intrinsic immunomodulatory mechanisms at the genetic or epigenetic level and may have carcinogenic pathogenicity (Table 2).

### 3.1. Streptococcus bovis

*Streptococcus bovis* (*S. bovis*) is a species of Gram-positive bacteria commonly associated with infective endocarditis [142] and bacteremia in humans [143]. It has been reclassified as *S. gallolyticus.* Although not classified as a carcinogen by IARC at this time, numerous studies have shown a relationship between the subspecies *Streptococcus gallolyticus* and colorectal cancer (CRC) [131,132,133]. Whether the relationship between *S. gallolyticus* and CRC is causal, correlated, or coincidental, was previously controversial. However, key studies have demonstrated that *S. bovis* and subspecies have concomitant inflammatory factors that concentrate in the intestine in CRC and promote colorectal tumorigenesis and progression of normal colorectal mucosa to adenoma and CRC through induction of the adenoma–carcinoma sequence [132] (see Section 2.1). Kumar et al. demonstrate that *S. gallolyticus* actively promotes colon cancer cell proliferation and tumor growth using in vitro cell cultures and mouse models of CRC, suggesting that its presence is causal in CRC and not merely temporal or commensal [131]. 

*S. gallolyticus* subspecies are uniquely able to paracellularly cross a differentiated epithelium without inducing epithelial interleukin-8 or 1β responses, thus evading the immune response and allowing for progression. Additionally, the ability to form biofilms on collagen-rich surfaces is a key virulence factor of *S. gallolyticus*, thus associated with infective endocarditis and pre-cancerous sites with a displaced epithelium [144]. In the previously referenced study by Kumar et al., increased levels of β-catenin, c-Myc, and PCNA were observed in colon cancer cells following incubation with *S. gallolyticus.* Stabilization of β-catenin seems to be of crucial importance to the contribution of *S. gallolyticus* in oncogenesis, as knockout of β-catenin was shown to abolish its effect. The mechanism or feature by which *S. gallolyticus* might stabilize β-catenin is still unclear and is an ongoing subject of investigation [131]. The disruption of the E-cadherin/β-catenin complex affects cell–cell adhesion and the Wnt-signaling pathway establishing the conditions for metastatic spread [68,72].

Other key studies have shown a causal relationship between *S. gallolyticus* and CRC. A 2018 study using an in vivo mouse model in the setting of inflammatory bowel disease showed that pre-inoculation with *S. gallolyticus* led to larger tumor formation and induced more colonic obstruction. This study further demonstrated that *S. gallolyticus* selectively recruits tumor-infiltrating myeloid cells, including marrow-derived suppressor cells, tumor-associated macrophages, and dendritic cells, which can inhibit the competence of T-cells [145]. The authors concluded that *S. gallolyticus* creates an immune-suppressive milieu that promotes neoplastic development in inflammatory bowel disease by recruiting tumor-infiltrating immune cells.

A similar study using an in vivo mouse model performed in 2019 by Deng et al. showed pretreatment with *S. bovis* aggravated tumor formation in mice compared to mice with adenomas only or healthy mice. After studying the cytokine expression pattern, increased levels of inflammatory cytokines including interleukin 6 (IL-6), IL-1β, TNF, and others were detected. Flow cytometry showed that *S. bovis* recruits CD11ᐩToll-like receptor 4 (TLR-4)ᐩ myeloid cells inducing a suppressive immunity conducive to CRC [146]. Evidence suggests that TLR-4 is associated with tumor development and progression and has been identified in numerous cancers including glioblastoma [147,148]. 

Butt et al. questioned the idea that the association between *S. gallolyticus* was present pre-diagnostically in a 2018 case–control study paired with a prospective cohort. Testing antibody responses to *S. gallolyticus* proteins in pre-diagnostic serum samples, they observed a positive association between antibody responses to *S. gallolyticus* and CRC development in serum samples taken before clinically evident disease onset, suggesting that *S. gallolyticus* serology might serve as a new marker for risk of developing CRC [149]. Current research on *S. bovis/gallolyticus* focuses on evaluating various subspecies and their pathogenicity [150], and genome-based drug target identification for early detection [151].

### 3.2. Escherichia coli

*Escherichia coli* (*E. coli*) is a Gram-negative, facultative anaerobe commensal bacterium found in the human gut. This species lives in the mucous layer as a harmless commensal, interacting with the host in a mutualistic manner. However, specific *E. coli* strains with pathogenic traits are correlated to diseases such as CRC [134]. More than two-thirds of colorectal cancer patients carry colibactin-producing *E. coli* strains in their gut and the number of carriers is rising in the Western world [152]. In particular, polyketide synthase (*pks*) genetic island positive strains of cyclomodulin-positive B2 *E. coli* (*pks + E. coli*), also known as B2 *E. coli*, are particularly cytotoxic and have been associated with CRC for their ability to produce the bacterial cytotoxin colibactin [153]. Colibactin is produced by *pks + E. coli* to suppress competing bacteria. However, colibactin has shown carcinogenic properties by modifying the tumor microenvironment [154] by inducing double-stranded DNA breaks and cell cycle arrest. The G2-M DNA damage checkpoint pathway is then activated, effectively depleting the mismatch repair proteins MSH2 and MLH1 in colonic cells through T3SS-induced effector proteins [155]. *E. coli* has also been associated with the production of biofilms [156]. 

Colibactin has also been demonstrated to stimulate colon tumor growth by producing a senescence-associated secretory phenotype via increased p53 SUMOylation [157,158]. Paulina et al., in a study that examined somatic mutations at colibactin target sites of several thousand cancer genomes, revealed notable enrichment of colibactin-induced DNA double-strand breaks in an AT-rich hexameric sequence motif in colorectal cancers, suggesting evidence for the etiological role of colibactin in human cancer [159]. Even short-term exposure to colibactin-producing *E. coli* transforms primary colon epithelial cells and demonstrates gene mutations seen in CRC, leading to enhanced proliferation, Wnt-independence, and impaired differentiation [160]. Further evidence suggests severe tumor microenvironment alterations result from *pks + E. coli* infection, including epithelial–mesenchymal transition, laying the foundation for potential metastatic disease [161]. There are ongoing studies into the causal or commensal nature of the relationship between *pks + E. coli* and CRC.

### 3.3. Fusobacterium nucleatum

*Fusobacterium nucleatum* is a Gram-negative obligate anaerobe that is opportunistic and commonly located in the oral cavity [162,163]. Recently, *F. nucleatum* has been reported to be within the primary lesion site of the cecum and the rectum in patients with colon cancer [135]. The hallmark mechanisms of *F. nucleatum* are increased cell proliferation, tumor-promoting inflammation, and avoiding immune destruction [164,165]. *F. nucleatum* stimulates cell proliferation via a number of mechanisms, including (1) *F. nucleatum* FadA binding to E-cadherin to activate the WNT/B catenin pathway [166] and (2) interacting with the Toll-Like Receptor 4 to activate PAK 1, a protein that phosphorylates the B-catenin pathway [167,168]. These two mechanisms of cell proliferation may lead to a decrease in the TOX family, which has been found to be associated with metastasis [169]. Additionally, FadA is responsible for tumor-promoting inflammation by increasing interleukins (IL-6, IL-8, and IL-18) and increasing the expression of nuclear factor-KB factor (NF-κB) [166]. The inflammatory association with *F. nucleatum* has been found to be correlated with miR-135b overexpression in patients with colon cancer [170]. Lastly, *F. nucleatum* can promote an immunosuppressive environment by working as an adhesive due to the overexpression of Gal-GalNAc on the fusobacterium apoptosis protein 2 (Fap2) to bind TIGIT immune receptor to inhibit NK cell cytotoxicity and T cell actions [171,172]. In colon cancer patients, common characteristics included microsatellite instability, methylation phenotype of the CpG island, and mutations in BRAF and KRAS. An interesting study completed by Guo et al. found that *F. nucleatum*-infected cells promoted tumor metastasis by the exosomes delivering miR-1246/92B-3p/27a-3 and CXL16/RhoA/IL-8 to non-infected cells [173].

### 3.4. Salmonella enterica

*Salmonella enterica*, a Gram-negative bacterium, is associated with colon cancers through two distinct proteins: the typhoid toxin, cyclomodulin, and AvrA [31,136]. AvrA is internalized via the type 3 secretion system leading to DNA damage, increased cell proliferation, and migration. AvrA activates the WNT/β-catenin pathway, increases STAT3 signaling pathway, and targets p53 [31,136,174]. STAT3 is a known promoter of tumorigenesis and therefore has a high potential to lead to CRC [175]. Cyclomodulin’s role in facilitating CRC is through its ability to suppress the secretion of various cytokines [31,136]. In addition, *S. enterica* has been found to initiate the MAPK/APT pathway which modulates cell proliferation [136]. Cytokine IL-22 is known to play a protective role in the abdomen, but a recent study illustrates that it may also facilitate *Salmonella’s* entry into the colon [176,177]. Excessive IL-22 also was found to inhibit apoptosis and promote tumor growth [178]. Therefore, it may contribute even further to the development of CRC. 

### 3.5. Enterotoxigenic Bacteroides fragilis

Enterotoxigenic *Bacteroides fragilis* (ETBF) is a strain of an anaerobic bacterium, *Bacteroides fragilis. B. fragilis* is present in the intestinal mucosal layer as early as 10 days after birth [179]. The ETBF strain increases cell proliferation and tumor-promoting inflammation to promote colorectal cancer [137,138,139]. This connection with colorectal cancer is through the ETBF association with inflammatory bowel disease, which may act as a precursor to colorectal cancer [180]. The ETBF strain secretes a *B. fragilis* enterotoxin (BFT) that promotes T-regulatory lymphocytes to increase the response of Th17 lymphocytes and increase IL-17 [181,182,183]. This increase in IL-17 activates the NF-κB pathway in the colonic epithelium, which leads to the secretion of chemokines that recruit myeloid-derived suppressor cells that ultimately favor tumor evasion of the immune response [184,185,186]. The STAT3 pathway has also been shown to be associated with the BFT toxin to promote proliferation and decrease apoptosis [187].

### 3.6. Enterococcus faecalis 

The literature is mixed regarding the role that *E. faecalis*, a Gram-positive bacteria, plays in the development of CRC. Some studies indicate that *E. faecalis* has no role in the development of CRC as seen by Viljoen et al. [140] who found no significant difference in *E. faecalis* levels between healthy patients and those with CRC. Other studies have recognized some protective and beneficial mechanisms of *E. faecalis* [188]. Miyamoto et al. [188] demonstrated that heat-killed *E. faecalis* suppresses polyp formation by weakening β-catenin signaling.

However, a large majority of studies have found a significant correlation between high levels of *E. faecalis* and patients with CRC [189,190,191]. The proposed mechanism through which *E. faecalis* promotes CRC is through its ability to produce reactive oxygen species and damage DNA [192,193]. *E. faecalis* can lead to DNA modification through its ability to induce aneuploidy and tetraploidy in colonic epithelial cells [194]. Another mechanism that has been proposed is through the activation of Wnt/β-catenin. Wang et al. [195] demonstrated an increased Wnt3α expression and suppressed Wnt inhibitor factor 1 (Wif1) leading to activation of Wnt/β-catenin after exposure to *E. faecalis*. *E. faecalis* may also lead to a proinflammatory state driven by the activation of MAPK and the conversion of macrophages to an M1 phenotype [196,197].

### 3.7. Clostridium septicum 

*Clostridium septicum* (*C. septicum*) is a Gram-positive, anaerobic, spore-forming bacillus found in the intestine and is a rare cause of gas gangrene that is associated with underlying malignancy in over 80% of cases [141]. It is linked to CRC and immunosuppression [198]. Infection with *C. septicum* may vary in manifestation and is associated with high mortality rates within 24 h if diagnosis and appropriate treatment measures are not immediately taken [199]. It is unclear if the association between *C. septicum* and CRC is associative or causal. It has been proposed that the hypoxic, acidic microenvironment of aggressive tumors via anaerobic glycolysis supports germination of *C. septicum* spores [200]. This explanation would suggest a non-causal association. Perforation of the gastrointestinal or colorectal epithelium might allow *C. septicum* spores to enter the blood causing sepsis [201,202]. *C. septicum* produces several exotoxins, including alpha-toxin, an essential virulence factor [203].

## 4. Tumor Microenvironment

Current cancer research emphasizes the importance of analyzing a tumor’s microenvironment. The complexity of these environments has led to a drastic shift in how science thinks about these tumors and the recognition of tumor microenvironments as organs [204]. Overall, there are specialized cells in a tumor microenvironment, including cancer-associated fibroblasts, endothelial cells, pericytes, tumor-associated macrophages, cancer stem cells, cancer cells, immune-inflammatory cells, and invasive cells [204,205]. All of these elements comprise the tumor’s habitat in which it can grow and thrive in the human body. Interestingly, inflammation has been noted to supply bioactive molecules (growth factors, survival factors, and extracellular-modifying enzymes) to the tumor microenvironment to allow angiogenesis, invasion, and metastasis of the tumor [128,206,207,208]. Marongiu et al. [209] described how phage-induced bacteriolysis drives inflammation through the release of cellular debris into the microenvironment. This change in the microenvironment is followed by a pathogen-associated molecular pattern to further stimulate the immune environment. This phage-induced mechanism changes the microenvironment which allows it to have the ability to alter the microenvironment and favor metastasis in the colon. These tumor microenvironments have also been noted to change as the tumor progresses to a metastatic tumor. The metastatic tumor microenvironment houses a plethora of invasive cancer cells in order to complete the seeding and implantation of the foreign tissue.

Tumor microenvironments found in colorectal cancer have been suggested to follow a conceptual model called “the bacterial driver–passenger model”, which describes the chronological shift of the tumor microenvironment. This model specifies that the first change is from DNA damage, followed by malignant transformations, and then the outgrowth of the “passenger bacteria” by the “driver bacteria” [210]. The driver mutations most commonly seen in colorectal cancer include adenomatous polyposis coli (APC), CTNNB1, deleted in colorectal cancer (DCC), P53, KRAS, and myelocytomatosis oncogene (MYC) [211,212,213]. 

### Metastasis Site Microenvironment

In the context of the metastatic spread of cancer, one important question to be answered is whether the tumor microenvironment of the primary tumor is the same as the secondary tumor site after metastasis. Emerging metastasis research related to tumor microenvironments has begun to uncover examples showing similarities between metastasis sites. In a study performed by Fumagalli et al. [214], it was found that Leucine-rich repeat-containing G-protein coupled receptor 5-positive (Lgr5(+)) tumor cells were found to disseminate into the blood and were present at distant metastatic sites. A similar conclusion was drawn by Hirotsu et al. [215], who studied clonal and polyclonal seeding at distant metastasis sites. It was found that the clonal mutations were present in both tumor locations, creating similar tumor microenvironments at both the primary tumor site and the metastasis site.

The “Seed and Soil” hypothesis states that certain tumor cells can only successfully colonize selective organs which have suitable growth environments [216]. The current view of the “Seed and Soil” hypothesis consists of three important concepts. First, primary tumors and their metastases consist of genetically diverse tumor and host cells. Second, metastasis selects for cells that can succeed in all phases of the metastatic process. Lastly, metastases generally develop in a site-specific way. Since the microenvironments of each organ are different, individual cancer cells may be able to colonize one specific organ. However, this view of cancer organotropism is beginning to change in light of new evidence [217]. 

**Figure 4:**
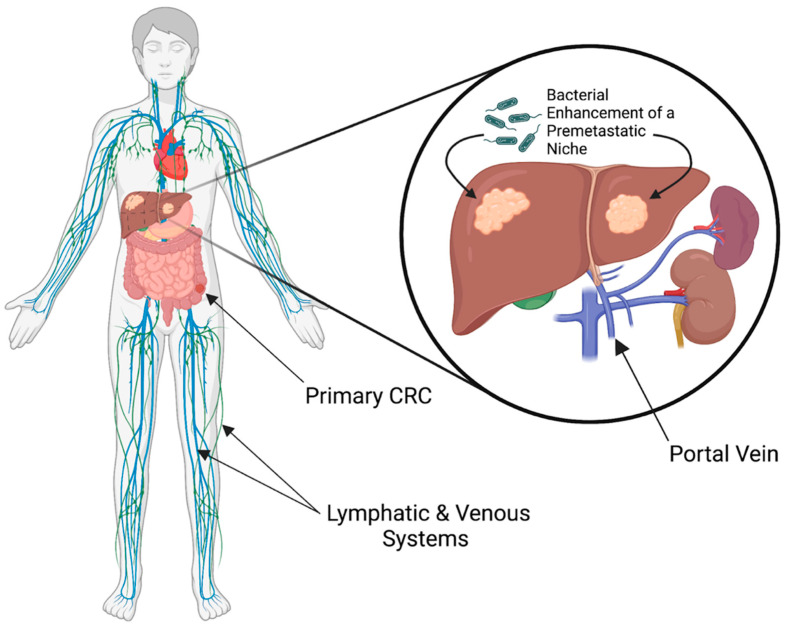
Bacterial influence in the metastatic cascade at the primary tumor and formation of a premetastatic niche at the site of metastasis. Figure created using biorender.

A better understanding of the process of metastatic spread of cancer and its several stages such as intravasation, extravasation, tumor latency, and development of metastasis has been defined in the last decade. Furthermore, recent studies have shown that the target organs may be prepared for metastatic deposits by the development of pre-metastatic niches, meaning that organs of metastasis are selectively modified by the primary tumor before metastatic spread has occurred [218]. The liver is the most common site of CRC metastasis, notwithstanding the significant difference in the microenvironment of the liver from the colon. Tumor cells, therefore, must either adapt themselves to a new environment, or the environment itself must change or be changed to be more favorable for tumor cell survival and growth. Evidence suggests the modification of the innate immune profile of the liver does occur, and that bacteria might be indicated in the modification. One proposed mechanism of modification is accumulation of hepatic natural killer T-cells by bacterial mediated regulation of bile acid metabolism (Figure 4) [219].

*S. bovis*, *C. septicum*, and *Peptostreptococcus* were illustrated to be enriched in the tumor microenvironments, possibly promoting CRC development [220]. Tjalsma et al. [8] conducted a study that found similarities between bacterial species that inhabit on- and off-tumor sites. Specifically, *Fusobacterium* and *Streptococcus* inhabit the tumor tissue samples, whereas *Salmonella* inhabits the surrounding tumor tissue sites. Another mechanism that is relevant to the discussion on colorectal cancer tumor microenvironments involves invasiveness linked to the gain of function of epithelial membrane protein 1. This highlights that direct cell-to-cell contact can induce gene expression within the tumor microenvironment to promote metastasis of colorectal cancers [221]. Ballman et al. [222] demonstrated similar preliminary findings in a 2017 human study that used PCR to compare primary and metastatic tumors, showing 64% positivity for similar strains of *Fusobacterium* at both sites in 11 patients. 

Marongiu et al. [209], in a metagenomic analysis of primary colorectal carcinomas and their metastases, analyzed whole-genome sequences of CRC primary tumors and their corresponding metastasis sites for sequences of viral, phage, and bacterial species. While multiple bacterial species were shown to be enriched at the primary tumor, only enrichment of *E. coli* strains was observed in metastasis sites. However, Bertocchi et al. showed that gut vascular barrier impairment leads to intestinal bacteria dissemination and colorectal cancer metastasis to the liver. Using endothelial marker plasmalemma vesicle-associated protein-1 (PV-1), they showed that migrated *E. coli* induce a pre-metastatic niche in the liver, favoring the deposition and proliferation of metastatic cancer cells. Furthermore, they found that in tumor-bearing mice, antibiotic treatment reduced liver polymorphonuclear neutrophils (PMN), suggesting that bacteria influence cancer cell survival and proliferation at metastasis sites [223].

Evidence suggests that bacteria may travel with circulating tumor cells into venous circulation and to metastatic sites such as the liver. Paired migration may occur passively through portal circulation, or actively through a quorum sensing type mechanism. Quorum sensing is a bacterial communication method that allows for self-organization into cooperative groups to carry out complex processes en masse, such as biofilm formation and colonization at remote locations [224]. *E. coli* and *E. faecalis* have been noted to use quorum-sensing peptides to facilitate organ specific homing, commonly selectively targeting the liver. Preliminary studies suggest that by emitting a chemoattractant agent to crosstalk with CRC tumor cells, these particular species may promote invasion and angiogenesis, enhancing liver-specific homing [225]. It has been hypothesized that cancer cells themselves may use quorum sensing mechanisms to influence one another [226]. 

There is evidence that tumor cells can lie dormant and undetectable within host organs, even for years, before being re-awakened and beginning uncontrolled proliferation [227]. It has been hypothesized that bacteria may hasten or enhance the process of reactivation through local or systemic inflammation and neutrophil recruitment, although this has not been confirmed in human models [228]. 

A biofilm is a collection of surface-associated bacteria enclosed in an extracellular polymeric substance matrix. Biofilm adhesion disrupts the mucous layer of the colon and allows for cyclomodulin-mediated epithelial DNA damage [156]. CRC initiation and progression often involve organization of bacterial communities into biofilms [229]. In fact, mucosal-invasive bacterial biofilms are identified on the colon mucosa of approximately 50% of colorectal cancer (CRC) patients and approximately 13% of healthy subjects [230]. In a 2019 mouse study, Tomkovich et al. [231] showed that mucosal biofilms, whether from tumor hosts or healthy individuals undergoing screening colonoscopy, are carcinogenic in murine models of CRC. *S. bovis* and *E. coli* have been noted to form biofilms as part of their pathological modus operandi [144,156]. Biofilms and the above-described mechanisms are identified with their associated bacteria in Table 3.

Few published experiments include other sites besides the liver to draw comparisons between the tumor microenvironments and bacterial colonization after colorectal cancer metastasis, likely because the liver is by far the most common site of metastasis. Marongiu et al. [209] found no bacterial species in lung metastases of CRC. However, a relationship between gut microbiome dysbiosis and lung cancer has been established [232], suggesting that similar mechanisms may be at play. An increasing amount of literature shows important relationships between the gut microbiome and physiological processes in the body that involve anatomic sites of CRC metastasis such as brain [233], bone [234], and lung [232].

Further investigation into the comparison of the microbial profile and microenvironment of the metastatic site and primary tumor may yield further points of possible intervention to prevent the deadly proliferative spread of colorectal cancer, and we should not limit investigation to the liver for these reasons. Epithelial–mesenchymal transition, and the influence of bacteria on this key metastatic step should be further investigated. Specific effects of bacteria on the various patterns of metastasis including venous and lymphatic spread is another area of study that lacks significant evidence. Additionally, investigation into the timing of bacterial action in relation to the metastatic cascade or metastatic proliferation to better guide future approaches to targeted treatment attempts is warranted. While the concept of bacterial inflammation promoting neoplasia has been well established, further study on the interrelationship between bacterial infection, inflammation, and metastasis is indeed needed to characterize the mechanisms at play. There are no studies to show preferential interference of specific bacteria in the high and low MSI or CIN pathways, and studies in this domain could be beneficial. Tumor cell dormancy and bacteria-induced inflammation-mediated reactivation should be investigated, including how to detect and mitigate this process early. Beyond anatomical considerations, investigations must move towards better understanding of molecular players in cancer metastasis and organotropism and particularly how the microenvironment is altered, such as by microbes, in order to exploit these processes and avoid their fatal result.

## 5. Microbial Considerations in Colorectal Cancer Treatment

Treatment of colorectal cancer requires broad consideration of many factors, such as specific organ region involvement, tumor staging, tumor cell markers, and current pharmacotherapy options. Prognosis is more accurately determined after thoroughly investigating the cells and environment. Right-sided colon cancers have a generally worse prognosis, in part due to these cancers generally garnering more somatic mutations [115]. Mutations in the oncogene NRAS respond poorly to anti-EGFR treatment and therefore have a poorer prognosis [235]; for this reason, the European Drug Agency does not recommend this treatment [236]. BRAF mutations also indicate a poor prognosis but are rare and usually found in the ascending colon of elderly females [235]. 

Many studies produced inconclusive results regarding whether CRC with BRAF mutations is responsive to anti-EGFR treatment, but it is generally not attempted [237,238]. KRAS mutations are more common in females, but studies are conflicted on whether they are associated with a specific region in the colon [44,235]. BRAF and KRAS mutations are mutually exclusive, occurring in 15% and 35% of sporadic CRC, respectively [49,239]. Binimetinib, Encorafenib, and Cetuximab triple therapy shows promise for being a new standard of care treatment for CRC with the BRAF mutation [240]. Although usually associated with breast cancers, HER2 mutations in CRC can occur and are responsive as expected to anti-HER2 treatment [235]. These sources show that mutations have already been extensively studied. 

Bacterial loads have been considered in treatment with significant results. One study showed that targeting colonic *F. nucleatum*, in addition to chemotherapy, may decrease treatment resistance to Oxaliplatin and 5-FU, thereby improving outcomes; patients with a high load of these bacteria show a poorer prognosis [168]. Enterotoxigenic *B. fragilis* is another bacterium that is particularly associated with left-sided carcinogenesis when in high abundance [241]. Targeting this microbe could further improve the more favorable prognosis of left-sided CRC, as well as decrease the occurrence of premalignant lesions [242]. The potential for use of microbial variation markers for non-invasive early diagnosis prognostic assessment of CRC and advanced adenomas is an ongoing area of investigation with promise [243]. 

Interestingly, bacterial biofilms have been highly associated with right-sided CRC; the reason for this is suspected to be decreased E-cadherin produced by the intestinal epithelium, leading to increased permeability and subsequent inflammation [244,245]. This creates the problem of dealing with biofilm accumulation to reduce CRC risk, despite the microbes inside being granted increased resistance to antibiotics. To further support the need to consider biofilms in treatment, Dejea et al. in human studies found that adenomas in patients with familial adenomatous polyposis were highly associated with biofilms that were notably colonized by the inflammatory microbes *E. coli* and *B. fragilis* [156]. Considering the relative populations of inflammatory flora of the patient’s colon will help create personalized treatment plans that would be then expected to increase patient survival outcomes.

## 6. Synthesis and Conclusions

Colorectal cancer is a major global cause of morbidity and mortality. Primary tumors, if caught early, are often treatable, but metastatic disease remains a challenging clinical problem and is present in the majority of cases of cancer-caused mortality. Bacterial involvement in colorectal neoplasia and metastasis is significant. The most common site of CRC derived metastasis is the liver. It used to be held that liver metastasis selectivity was simply a matter of anatomic relationship via portal vein hematogenous spread. However, increasing evidence suggests that bacterial infection not only promotes carcinogenesis in primary CRC, but also affects metastatic progression and organotropism through modifying the microenvironment at primary and secondary tumors. The specific mechanism by which bacteria modifies the metastatic microenvironment needs investigation. Bacterial virulence factors induce inflammation and disruption of epithelial integrity, which allows for the primary tumor to undergo the key steps of the metastatic cascade. Paired migration and quorum sensing are processes by which bacteria self-signal, recruit, and effectively establish a pre-metastatic niche at distant sites, rendering a suitable environment for tumor cell survival and proliferation. Further investigation into exploiting these processes through targeted antibiotic therapy to interrupt progression is supported by preliminary studies.

## Figures and Tables

**Table 1:** Colorectal neoplasia classifications and their most common metastatic sites.

Neoplasia Classification	Most Common Site of Metastasis	References
Adenocarcinoma	Liver	[13]
Carcinoid	Liver	[15]
Lymphoma	Spleen, bone	[21]
Gastrointestinal stromal	Peritoneum, liver	[16]
Leiomyosarcoma	Liver, lung	[17]
Melanoma	Liver, lung	[18]
Squamous cell carcinoma	Liver	[19]

**Table 2:** Overview of bacteria associated with colorectal neoplasia.

Bacteria	Proposed Pathogenesis	References
*Streptococcus bovis* (subspecies *gallolyticus*)	Increased cellular proliferation and signaling	[131,132,133]
*Escherichia coli*	Genomic instability (DNA damage), promoting inflammation, and epithelial–mesenchymal transition	[134]
*Fusobacterium nucleatum*	Immunosuppression, inflammation, increased cellular proliferation	[135]
*Salmonella enterica*	Transformation, inflammation, increased cellular proliferation	[31,136]
*Enterotoxigenic Bacteroides* *fragilis* (ETBF)	Increases cellular proliferation and tumor growth. Disrupts cytoskeleton by binding to E-cadherin	[137,138,139]
*Enterococcus faecalis*	Genomic destabilization	[140]
*Clostridium septicum*	Myonecrosis, sepsis	[141]

**Table 3:** Bacterial involvement in colorectal neoplasia progression and metastasis according to the steps of the metastatic cascade and species-specific mechanism of action.

Metastatic Cascade Step	Mechanism	Species	Reference
Detachment and invasion	β-catenin stabilization	*S. bovis* *F. nucleatum* *S. enterica*	[131]
	Induction of epithelial– mesenchymal transition	*E. coli*	[134]
	NF-κB activation	*F. nucleatum* *B. fragilis*	[166]
Intravasation and dissemination	Biofilm	*S. bovis* *E. coli*	[144] [156]
	Quorum sensing	*E. coli* *F. nucleatum*	[225]
Extravasation and proliferation at distant site	Paired migration	*E. coli*	[223]
	Local microenvironment alteration	*E. coli*	[225]
Dormancy	Tumor cell reactivation	Undetermined	[228]
